# Progress in retinal toxicity research

**DOI:** 10.1007/s00204-022-03222-4

**Published:** 2022-01-31

**Authors:** Hermann M. Bolt, Jan G. Hengstler

**Affiliations:** Leibniz Research Centre for Working Environment and Human Factors at TU Dortmund (IfADo), Adeystr. 67, 44139 Dortmund, Germany

Retinotoxicity of chemicals has been an issue for the Archives of Toxicology since decades. Already in the 1930s, we published case reports on amblyopy caused by intoxications, e.g., by arsenicals (Leinfelder 1938) and methanol (Menne 1939). At that time, arsenicals were still ingredients of a number of medicines.

Much later, significant advance of diagnostic tools, such as electroretinographic assessment of early retinopathy in rats (Maertins et al 1993), triggered a new era of research on retinotoxins, by combining morphological and physiological methods (Nyska et al 1992). Examples of toxicants examined are 2-hexanediol (Bäckström et al 1993, 1998; Nylen et al. 1993) and the antibacterial agent sitafloxacin (Shimoda et al 2001).

Currently, we witness further significant advancements in analytical tools and molecular biology. For instance, Wright et al (2019) used RNA profiling to determine the distribution of genes in the human retina, in relation to retinal toxicity. Other topics of publications were the influence of blue light (Alaimo et al 2019), and degeneration of retinal pigment epithelial cells by the toxic fluorophore *N*-retinyl-*N*-retinylidene ethanolamine (Alaimo et al 2020).

In general, ocular toxicity remains as an important aspect in preclinical testing, and refinements of the general strategy are warranted.

In this issue of the Archives of Toxicology, Hamm et al (2022) from AstraZeneca, U.K., focus on the receptor tyrosine kinase, MERTK, which plays an essential role in homeostasis of the retina, via efferocytosis of shed outer nuclear segments of photoreceptors. MERTK is a receptor tyrosine kinase and part of the TAM family that is involved in regulation of the innate immune response. There is much interest in harnessing the potential of MERTK as a novel oncolytic therapeutic target. However, a significant body of evidence shows that genetic mutation of MERTK is associated with retinal degeneration in rats and mice. Moreover, this phenotype translates to human loss-of-function mutations of MERTK which result in *retinitis pigmentosa*. Hamm et al (2022) describe a pre-clinical strategy to investigate the potential for ocular toxicity of a novel and selective MERTK inhibitor, which involved the application and integration of state of the art technologies, including multi-modal MSI, histopathology and EM. This approach enabled in-depth characterisation of an ocular lesion at both a molecular and morphological level. Furthermore, back-translation to an in vitro human retinal mode was assessed. The strategy appears equally applicable to characterise other small molecule inhibitors with a risk of ocular toxicity, which appears to be of relevance with the increasing prevalence of clinical ocular toxicity, particularly for tyrosine kinase inhibitors.

Further contributions to this topic to the Archives of Toxicology are highly welcome!
